# Seroprevalence and risk factors for sheep pox and goat pox among small ruminant herds from Nakapiripirit and Sembabule districts, Uganda

**DOI:** 10.3389/fvets.2025.1579164

**Published:** 2025-07-25

**Authors:** Nizeyimana Gerald, Ayebazibwe Chrisostom, Biryomumaisho Savino, Mayanja Franklin, Muhanguzi Dennis, Vudriko Patrick, Tweyongyere Robert, Erume Joseph

**Affiliations:** ^1^Department of Pharmacy Clinical and Comparative Medicine, School of Veterinary Medicine and Animal Resources, College of Veterinary Medicine Animal Resources and Biosecurity, Makerere University, Kampala, Uganda; ^2^National Animal Disease Diagnostics and Epidemiology Centre, Directorate of Animal Resources, Ministry of Agriculture, Animal Industry and Fisheries, Entebbe, Uganda; ^3^Department of Biotechnical and Diagnostic Sciences, School of Biosecurity, Biotechnology and Laboratory Sciences, College of Veterinary Medicine Animal Resources and Biosecurity, Makerere University, Kampala, Uganda

**Keywords:** cross-sectional, sheep pox and goat pox, seropositivity, risk factors, antibodies

## Abstract

**Background:**

Sheep pox and goat pox diseases[SP&GP] caused by sheep poxvirus and goat poxvirus, respectively are transboundary, World Organization for Animal Health class A-notifiable viral diseases. In Uganda, despite the inevitable national underreporting, suspected sheep pox and goat pox outbreaks have been reported from passive surveillance. There are limited sheep pox and goat pox serological data in small ruminants in Uganda.

**Materials and methods:**

A cross-sectional study was conducted in Sembabule and Nakapiripirit Districts, largely representing semi-intensive and communal/pastoral small ruminant production systems, respectively, between May and September 2023 to (i) determine the seroprevalence of sheep pox and goat pox virus antibodies in goats and sheep and (ii) identify the risk factors for the spread and transmission of SP&GP in small ruminant herds. A total of 1,515 serum samples were collected from 63 farms/clusters in Sembabule [*N* = 703] and Nakapiripirit [*N* = 812] districts and analyzed using Double Capripox multispecies antigen ELISA test to detect SP&GPV antibodies. Univariate and bivariate statistical analyses for animal and farm/cluster level factors were performed for significance using chi square and Fisher’s exact tests, respectively.

**Results:**

Of the total samples tested, [*n* = 137, 10%; CI, 8.5–13.1%] were positive for S&GP antibodies. Seropositivity was greater in Nakapiripirit [*n* = 93, 12%] than in Sembabule districts [*n* = 44, 6%]. Although low in proportion, overall, from both districts, seropositivity among sheep [N = 296, *n* = 32,12%] was higher than goats [*N* = 1,219, *n* = 105,10%]. Age, sex, type of epidemiologic unit, gifting of animals and species (*p* < 0.05) were associated with S&GP seropositivity. However, at multivariable level, only age [OR (95% CI), *p*-value: 0.43 (0.21–0.87), 0.019], and sex [OR (95% CI), *p*-value, 2.14 (1.31–3.5), 0.003] were independently associated with S&GP seropositivity.

**Conclusion:**

This study confirmed exposure to SP&GPV in goats and sheep to varying degrees in the districts studied. We recommend, based on evidence, an extended nationwide evaluation of antibody seroprevalence in goats and sheep to inform national-level SP&GP control efforts. To the best of our knowledge, this is the first documented systematic study to evaluate the seroprevalence of S&GPV antibodies in sheep and goats in the two districts.

## Introduction

Livestock diseases in Uganda continue to threaten the realization of the agro-industrialization agenda as an economic pillar under the National Development Plan (III) (1). This is in addition to directly affecting the livelihoods of communities that depend on livestock. Keeping of sheep and goats in Uganda is often termed keeping cash on four legs, also referred to as cash accounts. Goats and sheep are affected by a host of diseases, including sheep pox and goat pox virus disease, affecting the primary source of livelihoods of dependent communities (2).

Sheep pox and goat pox virus diseases are caused by two virus strains closely related to each other and belong to the genus *Capripoxvirus*, subfamily *Chordopoxvirinae*, and *family Poxviridae* (3). Sheep pox and goat pox viruses are transboundary, World Organization for Animal Health (WOAH) class A-notifiable viral diseases of small ruminants endemic to African countries above the Equator, parts of the Middle East and Asia (3, 4). As stated, sheep pox and goat pox belong to the sub family Capripox viruses that are large complex linear double stranded DNA viruses (39). These two diseases present a real challenge to the livestock industry as they affect small ruminants and are often clinically confused due to overlapping clinical signs requiring additional laboratory confirmation (5).

Like most small ruminant diseases, SP&GP continue to keep the majority of Sub-Saharan Africa in poverty, where a substantial proportion of households depend on small ruminants for food [meat and milk] resources and income (6–8). In Uganda, the positive trend in the small ruminant population shown by recent statistics released in 2024, with numbers of up to 17.4 and 4.4 million goats and sheep, respectively, may not contribute to economic development unless the burden of small ruminant diseases is addressed (9). SGP presents with high fever and generalized macules that progressively become papules or skin necrotic lesions (10). The disease presents with postmortem nodular lesions of internal organs (10, 11). Morbidity due to SGP varies greatly from01 to 90% depending on the breed and endemic status, with imported breeds and naïve flocks being the most susceptible (12). Young and naïve flocks suffer the highest case fatality rate of up to 100% (13). Emerging markets, including live animal trade and uncontrolled animal movements in pastoral and agropastoral communities, are responsible for the spread of infectious animal diseases [Akwango, Quan, and Byaruhanga 2022; (14, 15)].

SP&GP is spread directly and indirectly through contact with infected animals, aerosols of nasal secretions, infected saliva, dried scabs, fomites and transportation vehicles (16). SP & GP-associated losses include multiples ranging from mortalities to reduced productivity in the form of market value, case management costs and distorted international trade in both live animals and animal products (3, 14, 15, 17, 18). Vaccination against SGP has proven to be a cost-effective approach at the herd level, although it is not religiously practiced in most developing countries (14).

Currently, there is a lack of up-to-date information on the seroprevalence of SP&GPV in small ruminant herds in cattle corridor where small ruminants are concentrated as well as associated risk factors in Uganda, despite its significant impact on small ruminant farming. We determined SP&GP seroprevalence based on antibody detection via double Capripox multispecies antigen enzyme-linked immunosorbent assay (ELISA) as well as -associated risk factors in the Sembabule and Nakapiripirit Districts representing two contrasting two small ruminant production systems, i.e., agropastoral and pastoral systems, respectively. The obtained data provided baseline serostatus as well as possible predictors for SP&GP spread among small ruminant herds that are crucial for implementing sheep pox and goat pox control measures.

## Materials and methods

### Description of the study area

The study was conducted in the districts of Nakapiripirit in northeastern Uganda [Karamoja] and Sembabule in central Uganda. The districts are in mid/central and northeastern part of the cattle corridor also referred to as the livestock dense corridor. The cattle corridor has been traditionally known as such in Uganda as a region stretching from Southwestern Uganda to Northeastern Uganda (9, 19). The corridor is predominantly semi-arid characterized by extensive savannah grasslands, scattered shrubs and acacia woodlands. This region predominantly supports livestock, and cattle has been used to synonymously refer to as livestock that includes sheep and goats (20).

Nakapiripirit district in Karamoja region represents a pastoral and extensive livestock production system, whereas the Sembabule district in Western Uganda represents an agro-pastoral, semi-intensive and commercial production system. The study population comprised goats and sheep in the two selected districts that belong to the cattle corridor, that predominantly keep livestock and receives 300–800 mm of rainfall, making them prone to shortages of pasture and water.

Nakapiripirit district has goat and sheep populations of 156,962 and 57,904, respectively, whereas the Sembabule district has estimated goat and sheep populations of 128,261 and 25,271, respectively (9). The two districts were selected based on the contrast in their production systems. Nakapiripirit in Karamoja specifically has stable herds compared with other districts because of low levels of livestock rustling and the infant steps toward sedentalisation. This was in addition to the lack of history of vaccination against SP&GP based on national records at the time of study, willingness and support from the district local governments. Sembabule, on the other hand, was selected because of the substantial small ruminant population, including a high number of exotic small ruminants, especially with the national goat improvement project based in Sembabule (9). In both districts, small ruminant records were extremely scarce, and as such, the questionnaire provided the most reliable information on the vaccination status in goats and sheep in addition to prior checks before embarking on the study. In Uganda up to 95% of the goat and sheep breeds are indigenous comprising of the small East African, Mubende and Kigezi goats (21), while for sheep up to 99.2% are indigenous and 0.8% exotic (9, 22). Goats and sheep production systems are predominantly extensive with very few shoats’ semi-intensive farms predominantly in the cattle corridor. In this manuscript, cattle corridor has been used to refer to the extensive savannah grasslands in Uganda where majority of the livestock are reared.

### Study design

**Figure 1:**
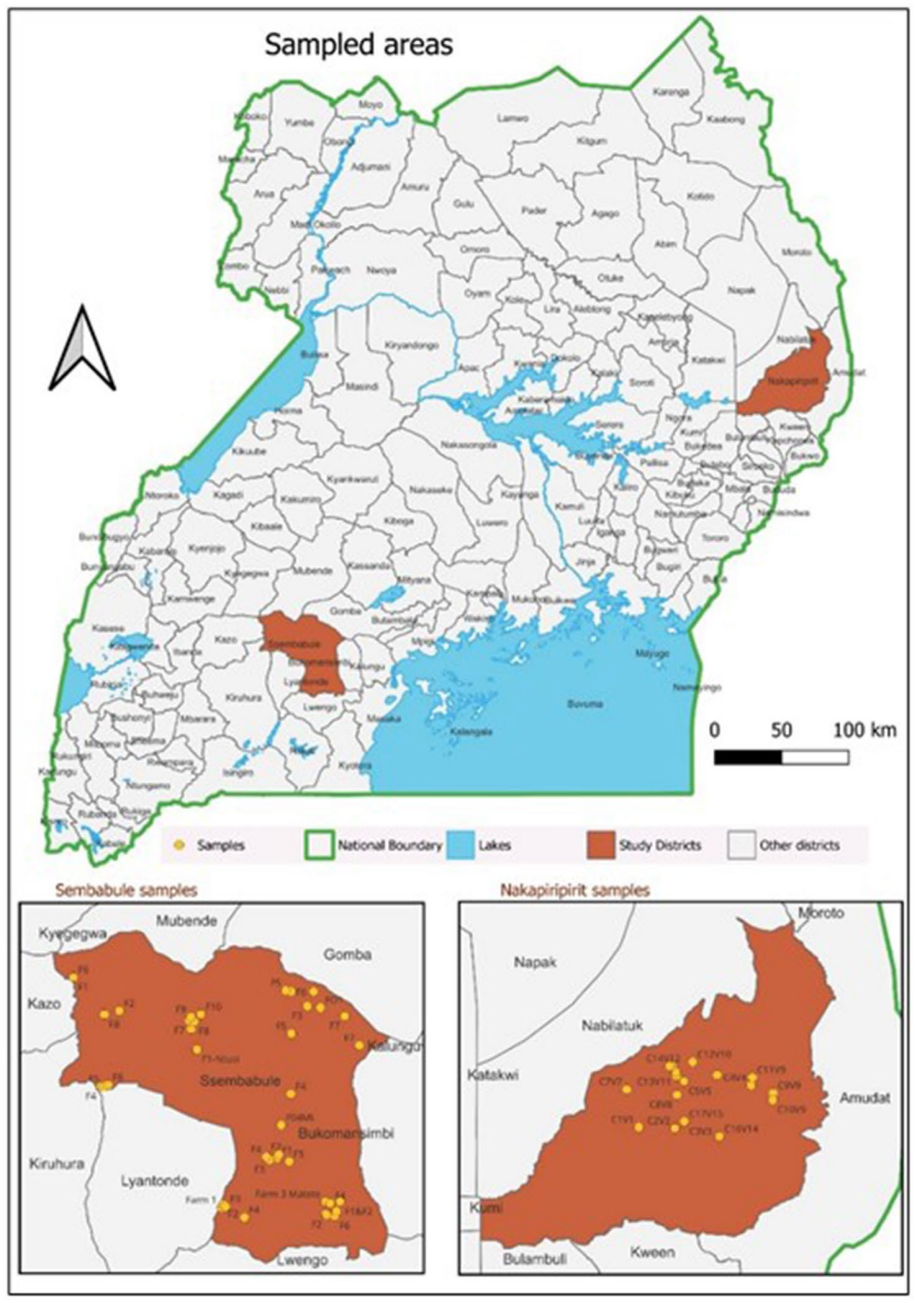
Locations of the sampled areas.

A cross-sectional study was designed to determine the SP&GP seroprevalence rate and associated risk factors from May to September 2023 in Sembabule and Nakapiripirit districts located in Central Uganda, and Northeast Uganda. A multistage sampling approach at the district, village and farm/cluster levels was used. The farms and clusters sampled are shown in Figure 1.

### Sample size determination

In addition to the minor exceptions of the farming system in Sembabule, where small ruminants are restricted to specific grazing units, in relation to Nakapiripirit, the village was considered the main epidemiological unit and, as such, informed the sampling frame in the study districts. The required number of villages/clusters on assumed estimated herd-level seroprevalence of 50% and a 95% confidence interval in target herds [farms and clusters] and an inter-cluster coefficient of 0.0289 and a design effect of 1.84 on Guillaume et al. (23), as implemented online,^1^ was 27 villages with an average number of samples of 30 to satisfy the set precision [up to 810 samples] per district. On the basis of the determined sample size, at the herd level, the minimum number of animals required to detect the presence of disease in a large population above 200 was 11, assuming that the expected disease occurrence at the animal level was 26% at the 95% confidence level, using Win Episcope 2.0 http://www.winepi.net/uk/sample/indice.htm implemented online (24). The SP&GP seroprevalence estimate was based on previously published estimates ranging from 15 to 31.47% (12, 25).

### Study village selection

A list of villages for the two districts as of 2022, accessed from UBOS as the sampling frame, was generated. The sampling frame was sorted alphabetically and assigned numerical numbers using Microsoft Excel. Unique random numbers equivalent to 30 villages per district [60 in total] were generated without replacement using the online randomizer.^2^ The random villages generated were traced back to the sampling frame to obtain the village details, including the parish and subcounty. Data capture was using a semi-structured questionnaire administered to the small ruminant farming households in the Nakapiripirit and Sembabule districts. A snow balling approach up to the required sample size in each of the selected villages was employed because of the lack of up-to-date farmer register. Information on the animal husbandry system, sheep pox and goat pox control practices, and potential risk factors such as herd size, grazing system, and vaccination status of small ruminants was collected. The Questionnaire was pretested and administered to sheep/goat herders and owners representing selected farms/clusters in the study districts. The questionnaire data was complemented with individual animal data collected on sample collection tubes and field forms. Obtained data was used to explain potential risk factors to sheep pox and goat pox sero status in the study areas.

This information was used to assess the possible risk factors for sheep and goat pox seropositivity in the study areas.

### Household and individual animal selection

Three households/farmers per village were selected, and a total of 10 animals per household were sampled [total, 30]. Probability proportional to population size in both districts was used, where in each of the selected villages, five and eleven [sheep] were to be sampled in Sembabule and Nakapiripirit, respectively. Using systematic random sampling, study animals were selected from village herds or from three households randomly selected from villages with individually owned flocks grazing in an area with a radius of approximately 5 km to minimize the number of sampling animals that share grazing areas and/or from the same herds.

### Inclusion and exclusion criteria

In the study districts, only sheep and goats above 6 months of age were considered and thus were sampled. Aging of the animals was based on records where available and actual physical aging using dentition. This age bracket was specifically selected because of the need to rule out any maternal SP&GP antibody transfer in goat and sheep kids younger than 6 months of age from the already selected villages in the study districts.

### Sample collection and transportation

Serum samples were collected from healthy goats and sheep above 6 months using the jugular vein into a 10 mL vacutainer via a 21-gage sterile needle. Using the area designated for identification on the vacutainer, each sample was identified by number, sex, age, and farm/cluster numbers. All the serum samples were left overnight at room temperature to separate the serum from the clotted cells. Each sample was aliquoted into a 2 mL cryovial and transported in duplicates on ice to the National Animal Disease Diagnosis and Epidemiology Center (NADDEC) and Research Center for Tropical Diseases [RTC] at Makerere University for storage at −20°C until analysis.

### Laboratory analysis

All the serum samples were tested using double Capripox antigen multispecies ELISA test kit [ID Screen®, ID vet, Garbles, France] for the presence of SP&GP antibodies against SP&GP V infection at NADDEC and RTC laboratory at Makerere University. 50 μL of each test serum sample was thawed and diluted in 50 μLs of dilution buffer 19 and added to an ELISA plate coated with purified Capripoxvirus antigen. Positive and negative control sera were similarly diluted and added to the designated wells of each ELISA plate. The ELISA plate was incubated for 90 min at room temperature, and the wells were emptied and washed five times with wash solution. One hundred microliters of conjugate were added to the wells, which were subsequently incubated for 30 min at room temperature. The wells were emptied and washed five times, 100 μL of substrate was added to each well, and the plate was incubated in the dark at room temperature for 15 min. This was followed by the addition of 100 μL of stop solution per well, and the optical density (OD) was read at 450 nm using a microplate reader (Biochrom Asys UVM 340, Cambridge, United Kingdom). For each sample, percentage positivity was calculated as the optical density of the sample minus the optical density of the negative control divided by the optical density of the positive control minus the optical density of the negative control multiplied by one hundred, represented in the formula below:


$$\frac{S}{P}\%=\frac{\text{ODsample}-\text{ODNc}}{\text{ODPC}-\text{ODNC}}\;X100$$

Tested serum samples with an S/P percentage of less than 30% were considered negative, and those with an S/P percentage greater than or equal to 30% were considered positive. Note: the test cannot differentiate SPPV and GTPV due to the high level of genetic similarity, and the result is thus referred to as SP&GP antibodies throughout this manuscript.

### Data management and statistical analysis

The field questionnaire and laboratory data were entered into Epidata version 3.2.2 before being exported to STATA version 16.1 for descriptive statistical analysis to determine the SP&GP seroprevalence and associated risk factors. The study area/areas sampled map was drawn using QGIS version 3.18 to portray the locations of the farms/clusters sampled. Seropositivity was determined by dividing the total number of antibody-positive samples by the number of animals sampled. Risk factors associated with the determined seroprevalence were analyzed with farm/cluster-level comparisons performed via Fisher’s exact test, whereas comparisons of animal-level factors were performed using the Pearson chi-square test for statistical significance. True antibody seropositivity was adjusted on the apparent seropositivity [AP], sensitivity [Se] (91%) and specificity [Sp] [99.7%] of the DCM-ELISA test Kit based on the formula by Stevenson, 2007 (26).

## Results

### Seroprevalence of sheep pox and goat pox

In the current study, out of 1,515 sheep and goat serum samples, 703 (Sembabule) & 812 (Nakapiripirit), analyzed with Double Capripox Multispecies Antigen ELISA, 137 [10%] tested positive for S&GP antibodies, with a 95% confidence interval [CI, 8.5–13.1%]. Nakapiripirit district had a higher seroprevalence [*n* = 93,11%] than Sembabule district [*n* = 44, 6%]. Although low in number, sheep were more positive than goats in both Sembabule [7, 6%] and Nakapiripirit [14, 12%], respectively (see Table 1). The demographic features of the study population were presented in Table 1. The median herd size was 32, and majority of the animals studied were females accounting up to 84.4% while 15.6% were males (Table 2).

**Table 1:** Showing SP&GP seropositivity, by district and species.

District	No. sampled	Sheep			Goats			Overall district seropositivity *N* (%)	True overall district seropositivity (%)
	(*N*)	# Sampled (*n*)	Apparent seropositivity *n* (%)	True seropositivity (%)	# Sampled (*n*)	Apparent seropositivity (%)	True seropositivity (%)		
Sembabule	703	112	8 (7%)	7	591	36 (6%)	6	44 (6%)	6
Nakapiripirit	812	184	24 (13%)	14	628	69 (11%)	12	93 (11%)	12
Overall	1,515	296	32 (11%)	11	1,219	105 (9.4%)	10	137 (9%)	10

*True Seropositivity (AP+Sp-1)/(Se+Sp-1) *100, Se [91%], Sp [99.7%], N Denotes number sampled at district level, *N* (%) Denotes, District level seroprevalence.

**Table 2:** Demographics and other key parameters of the studied farms/clusters.

Variable	Total (*N* = 63)
Herd size	
Mean (SD)	84 (94)
Median (Q1, Q3)	49 (32, 110)
District	
Sembabule	45 (71.4%)
Nakapiripirit	18 (28.6%)
Type of epidemiological unit:	
Cluster	16 (25.4%)
Farm	47 (74.6%)
Management system	
Pastoral system/communal grazing	29 (46.0%)
Semi-intensive	34 (54.0%)
Do you share grazing grounds and watering points with other farmers	
Yes	43 (68.3%)
No	20 (31.7%)
How often do you share farm equipment with other farmers in the neighborhood	
Always	5 (7.9%)
Sometimes	26 (41.3%)
Rarely	5 (7.9%)
Never	27 (42.9%)
Do you get advice on goat/sheep animal health husbandry?	
Yes	61 (96.8%)
No	2 (3.2%)
Since last year, have you experienced any sheep/goat deaths?	
Yes	55 (87.3%)
No	8 (12.7%)
Do you separate new sheep/goats brought in the farm?	
Yes	16 (26.2%)
No	45 (73.8%)
Are the vaccines and vaccination services for free?	
Yes	17 (27.0%)
No	46 (73.0%)
In your opinion, how do you rate your willingness to pay for vaccination	
Very willing	21 (33.3%)
Willing	29 (46.0%)
Not Willing	9 (14.3%)
Not sure	4 (6.3%)
In your opinion, how do you rate affordability of the vaccines for goats& sheep	
Affordable	17 (27.0%)
Somewhat affordable	25 (39.7%)
Not affordable	13 (20.6%)
Not sure	8 (12.7%)
In your opinion, how do you rate accessibility to the vaccines by goat and sheep	
Accessible	11 (17.5%)
Sometimes accessible	36 (57.1%)
Not accessible	9 (14.3%)
Not sure	7 (11.1%)

### Animal and farm level predictors of SP&GP seropositivity

Herd-and animal-level predictors of sheep pox and goat pox seropositivity revealed that sex [*p* = 0.005], age [*p* = 0.004], gifting from friends [*p* = 0.003] and the district of origin [*p* = <0.001] as well as the nature of the epidemiological unit [*p* = <0.001] were the main predictors of sheep pox and goat pox seropositivity (Table 3).

**Table 3:** Animal- and farm-level predictors of SP&GP seropositivity.

Variable	Sero-negativity	Sero-positivity	Total	*p* value
Age				
6–18 months	545 (93.3%)	39 (6.7%)	584 (38.5%)	**0.004**
>18–24 months	353 (91.9%)	31 (8.1%)	384 (25.3%)	
>24 months	480 (87.8%)	67 (12.2%)	547 (36.1%)	
Sex				
Female	1,151 (90.1%)	127 (9.9%)	1,278 (84.4%)	**0.005**
Male	227 (95.8%)	10 (4.2%)	237 (15.6%)	
District				
Sembabule	659 (93.7%)	44 (6.3%)	703 (46.4%)	**<0.001**
Nakapiripirit	719 (88.5%)	93 (11.5%)	812 (53.6%)	
Species				
Caprine	1,115 (91.4%)	105 (8.6%)	1,220 (80.5%)	0.228
Ovine	263 (89.2%)	32 (10.8%)	(295 9.5%)	
Management system	(N = 37)	(N = 26)	(N = 63)	
Communal/Pastoral system	20 (54.1%)	9 (31.0%)	29 (46.0%)	
Semi-intensive	17 (45.9%)	17 (53.1%)	32 (50.8%)	0.123
Epidemiological unit				
Cluster	2 (11.1%)	16 (88.9%)	18 (28.6%)	**<0.001**
Farm	35 (77.8%)	10 (22.2%)	45 (71.4%)	
Farmers sharing grazing grounds and watering points				
Yes	23 (53.5%)	20 (46.5%)	43 (68.3%)	0.276
No	14 (70.0%)	6 (30.0%)	20 (31.7%)	
Ever seen any animal(s) with Sheep and Goat Pox?				
Yes	4 (44.4%)	5 (55.6%)	9 (14.8%)	0.477
No	31 (59.6%)	21 (40.4%)	52 (85.2%)	
Farmer separates new sheep/goats brought in the farm?				
Yes	11 (68.8%)	5 (31.3%)	16 (26.2%)	0.381
No	24 (53.3%)	21 (46.7%)	45 (73.8%)	
Purchase from open market				
Yes	24 (52.2%)	22 (47.8%)	46 (73.0%)	0.094
No	13 (76.5%)	4 (23.5%)	17 (27.0%)	
From farm owners by breeding				
Yes	22 (55.0%)	18 (45.0%)	40 (63.5%)	0.596
No	15 (65.2%)	8 (34.8%)	23 (36.5%)	
Gifts from friends/relatives				
Yes	7 (31.8%)	15 (68.2%)	22 (34.9%)	**0.003**
No	30 (73.2%)	11 (26.8%)	41 (65.1%)	

*Farm/cluster-level comparisons were made using Fisher’s exact test, while animal-level factor comparisons were made using the Pearson chi-square test. *p values less than 0.05 were considered statistically significant. Bold indicates *p* values of statistically significant variables.

### Multilevel logistic model estimates of risk factors for SP&GP Seropositivity

At bivariate analysis, there was an association between SP&GP sero-positivity and: age, sex, type of epidemiologic unit, purchasing animals from open markets, receiving animals from friends and relatives as gifts, and species (*p* < 0.05). However, at multivariable level, only age and sex were independently associated with SP&GP sero-positivity. Male animals were less likely to be sero-positive than females with 57% lower odds of having SP&GP than the female animals [OR (95% CI), *p*-value: 0.43 (0.21–0.87), 0.019]. Older animals (>24 months) had at least 2 times odds of being S&GP sero positive than young ones (6–18 months), [OR (95% CI), *p*-value, 2.14 (1.31–3.5), 0.003; see Table 4].

**Table 4:** Multilevel logistic model estimates of risk factors for SP&GP Seropositivity.

	Un adjusted OR (95% CI)	*p* value	Adjusted OR (95% CI)	*p* value
Individual animal factors				
Age				
6–18 months	1.00		1.00	
>18–24 months	1.51 (0.87–2.64)	0.144	1.36 (0.78–2.39)	0.28
>24 months	2.43 (1.50–3.96)	**<0.001**	2.14 (1.31–3.5)	**0.003**
Sex				
Female				
Male	0.39 (0.19–0.78)	**0.008**	0.43 (0.21–0.87)	**0.019**
Species				
Caprine	1.00		1.00	
Ovine	1.74 (1.03–2.97)	**0.040**	1.51 (0.88–2.58)	0.134
Farm/cluster level factors				
Type of epidemiological unit				
Cluster	1.00		1.00	
Farm	0.15 (0.04–0.56)	**0.005**	0.26 (0.05–1.31)	0.103
Purchase from open market				
Yes	1.00		1.00	
No	0.22 (0.05–0.97)	**0.046**	0.49 (0.09–2.86)	0.432
Gifts from friends/relatives				
Yes	1.00		1.00	
No	0.25 (0.07–0.87)	**0.030**	0.57 (0.14–2.4)	0.448
Management system used				
Open/communal/Pastoral system	1.00			
Intensive/Semi-intensive	1.49 (0.44–5.11)	0.524		
Farmer shares grazing grounds and watering points with other farmers				
Yes	1.00			
No	0.81 (0.21–3.13)	0.763		
Farmer separates new sheep/goats brought in the farm?				
Yes	1.00			
No	1.29 (0.31–5.31)	0.726		

CI, Confidence interval.

## Discussion

Our study investigated the exposure of goats and sheep to S SP&GPV and associated risk factors in Uganda cattle corridor settings. Cattle corridor districts are also referred to as livestock dense districts. Overall, the seroprevalence of SP&GP was 9% in the two districts. The specific seroprevalence rates were 6 and 12% in Sembabule and Nakapiripirit districts, respectively. In terms of species, sheep had higher seroprevalence rates than goats at 12 and 9%, respectively. Our findings are consistent with those of another study in Ethiopia that reported higher seroprevalence in sheep than goats (12). These findings however contrasted the study in India that showed higher seroprevalence in goats than sheep (25).

In the absence of routine vaccination in the studied districts, sheep pox and goat pox antibodies are attributable to SP&GP natural infections in small ruminants. Importantly, by the time SP&GP antibodies are detected, the animal has recovered from clinical disease on the basis of S&GP pathogenesis where detection of SP&GP antibodies before 14 days of infection is not possible (2, 27, 28). Recovered animals obtain lifelong immunity from sheep and goat pox (28). The period from infection to the detection of SGP antibodies is important in animal movement control, where vaccination is necessary before authorization of movement (29). On the other hand, the proportion of animals protected based on the detected antibody levels was low; thus, urgent interventions are needed to protect herds by vaccination before SGPV incursion to small ruminants’ herds in the studied districts.

The presence of SP&GP antibodies among small ruminant herds further confirms the existence of S&GP in goats and sheep, as reported in previous reports of average, four outbreaks annually since 2011 (2). This observation strengthens the need to undertake a nationwide SP&GP seroprevalence study to map high-risk hot spots, gage the S&GP burden and inform control strategies. Under the current resource set up in Uganda, where resource allocation to veterinary services at local government and national level was low with less than 0.56 USD allocation per livestock keeping household (30), mass vaccination is not feasible but rather risk-based vaccination against SP&GP alongside other disease control measures that are equally logistically challenged.

Older animals above 2 years of age were more likely to be positive than young animals were [*p* value, 0.04], a finding that is consistent with that of Adeyinka et al. This could be because older animals were likely to be exposed to SP&GP infections for a longer period of time than young ones, suggesting an increase in susceptibility with increasing age (31). This however contradicts a study done in Ethiopia where young animals were likely to be positive compared to adult animals (32). Compared with males, females were more likely to be positive in the studied areas [*p* value, 0.05], possibly because males are usually sold earlier than their female counterparts since the latter are used for breeding and replacement stock, this was in addition to males being up to 57% less likely to be positive than females [OR 0.43 (0.21–0.87; Table 4)]. This finding agrees with the study done in Ethiopia in 2022 that indicated that females were 3 times likely to be positive than males (32). For all the farms, there were relatively few males, and as such, the number of females sampled was always greater because of their proportionality to size at the farms (12).

The seropositivity was greater in Nakapiripirit than in Sembabule districts, possibly because of persistent risky practices, such as constant comingling of herds in different communal grazing clusters, uncontrolled animal movements during periodic animal migrations in search of pastures and water, and overnight crowding in protected kraals to avoid animal rustling, among others. These factors facilitate direct spread to other animals (15, 28). In contrast, farms in the Sembabule district have transitioned to more semi-intensive production with improved care of animals, nutrition, and routine management of endoparasites, among others. Relatedly, majority farms and clusters shared grazing grounds, which is an important factor in SP&GP spread, although during the study, this factor was not a significant farm-level risk factor.

This study further revealed important risk factors, such as a lack of isolation facilities for newly introduced animals, with fewer than 20% of the farmers indicating that these facilities and regular animal exchanges among relatives as gifts were significant risk factors for SP&GP seropositivity [*p* value, 0.03]. The latter was particularly practiced for small ruminants owing to their size; they are easy to transport, do not routinely require veterinary inspection compared with cattle in practice, and are cheap in terms of value to give out for small ceremonies, among others. This practice poses risks not only for S&GP spread but also for other important small ruminant diseases, including zoonoses, as highlighted by Sherman (33), thus requiring due attention. This finding agrees with previous authors concerning the role of uncontrolled animal exchanges in infectious diseases of livestock spread in Uganda (34).

Among the farmers’ responses, especially those who had previously had SP&GP clinical infections at their farms, [9%] were able to recite the associated signs, including swellings on the skin, lumps and lesions on the skin and legs, nodules on the skin, papules on the ears and lips, pox-like lesions on bare parts of the body, swellings, fever and lameness. In the affected herds, those aged between 6 and 12 [55%] were more affected than those aged above 12 months [33%] and less than 6 months [11%], possibly because of maternal antibody transfer in young animals and the development of an immune system in adult goats and sheep (35, 36), indicating that the most important age category for vaccinating in the endemic setting was affected. Farmers had employed a range of measures, including (1) prophylactic treatment with antibiotics such as procaine penicillin and oxytetracycline and (2) isolation of infected animals to prevent contact as well as selling early all those in contact with those that showed signs also called panic sales to ameliorate the losses incurred. The latter practice has the potential to spread the disease to other farms, especially when animal movement regulations and enforcement are not robust, as was the case in the studied areas.

In terms of farm-level action to control sheep pox and goat pox, majority farmers up to 70% agreed that livestock vaccination improved the health of the animals. Up to 79% indicated a willingness to vaccinate sheep and goat pox, with up to 66% being able to afford the vaccine should it not be beyond 1,000 Uganda shillings per animal and readily available to save their economic and livelihood assets (Table 1). This is consistent with a study conducted in Ghana, where farmers were willing to pay for livestock vaccination as long as the unit price was within the affordable range (37). These findings paint a ray of hope in sheep and goat pox control because the current vaccine market price is within the affordable price range in addition to being directly symbiotic with Uganda’s policy shift, which requires farmers to pay for livestock vaccines. However, the farmers in Sembabule were more receptive to pay than those in Nakapiripirit, probably due to the level of advancement and commercialization in the small ruminant value chain; previous experience with sheep pox and goat pox mortalities in improved Boer and savannah goat breeds that are predominant in the district are critical incentives to Sheep pox and goat pox vaccination among farmers (14, 38).

Our study, however, had several limitations, including (1) the financial and laboratory capacity to perform a virus neutralization test, which is considered the gold standard test for SP&GP antibody detection, and (2) poor records, making it impossible to identify animals relying only on the memory of caretaker herdsmen, especially in Nakapiripirit, as well as insecurity, which interferes with access to some of the study areas.

## Conclusion

The study revealed an overall seroprevalence of sheep pox and goat pox of 11 and 10% in sheep and goats, respectively. The seroprevalence ranged from 7 to 11% and 6 to 10% among sheep and goats from in Sembabule and Nakapiripirit districts, respectively. Age, sex, epidemiological unit, gifting, and district studied were the main animal- and farm-level predictors to SP&GP seropositivity.

In the absence of routine vaccination against SP&GP, our results provide serological evidence of exposure to sheep and goat pox viruses in sheep and goat populations in the study areas. Systematic investigations, monitoring, and reporting of outbreaks are necessary to inform the design of control and preventive measures to prevent SP&GP -related economic, livelihood and welfare challenges.

## Data Availability

The raw data supporting the conclusions of this article will be made available by the authors, without undue reservation.

## Footnotes

^1^ https://web1.sph.emory.edu/users/cdckms/samplesize%20icc%20deff2.html

^2^ https://www.randomizer.org/
